# Standardized measurement of abdominal muscle by computed tomography: association with cardiometabolic risk in the Framingham Heart Study

**DOI:** 10.1007/s00330-022-08934-w

**Published:** 2022-07-02

**Authors:** Andreas Kammerlander, Asya Lyass, Taylor F. Mahoney, Jana Taron, Parastou Eslami, Michael T. Lu, Michelle T. Long, Ramachandran S. Vasan, Joseph M. Massaro, Udo Hoffmann

**Affiliations:** 1 Cardiovascular Imaging Research Center, Massachusetts General Hospital, Harvard Medical School, Boston, MA, USA; 2 Division of Cardiology, Medical University of Vienna, Vienna, Austria; 3 Department of Mathematics and Statistics, Boston University, Boston, MA, USA; 4 Department of Biostatistics, Boston University School of Public Health, Boston, MA, USA; 5 Department of Radiology, Medical Center - University of Freiburg, Freiburg, Germany; 6 Section of Gastroenterology, Evans Department of Medicine, Boston University School of Medicine, Boston, MA, USA; 7 Department of Internal Medicine, Boston Medical Center, Boston University School of Medicine, Boston, MA, USA; 8 Department of Epidemiology, Boston University School of Public Health, Boston, MA, USA; 9 The Boston University and the National Heart, Lung, and Blood Institute’s Framingham Heart Study, Framingham, MA, USA

**Keywords:** Muscle mass, Muscle fraction, Computed tomography, Cardiometabolic risk, Cardiovascular risk

## Abstract

**Objectives:**

To provide a standard for total abdominal muscle mass (TAM) quantification on computed tomography (CT) and investigate its association with cardiovascular risk in a primary prevention setting.

**Methods:**

We included 3016 Framingham Heart Study participants free of cardiovascular disease (CVD) who underwent abdominal CT between 2002 and 2005. On a single CT slice at the level of L3/L4, we segmented (1) TAM-Area, (2) TAM-Index (= TAM-Area/height) and, (3) TAM-Fraction (= TAM-Area/total cross-sectional CT-area). We tested the association of these muscle mass measures with prevalent and incident cardiometabolic risk factors and incident CVD events during a follow-up of 11.0 ± 2.7 years.

**Results:**

In this community-based sample (49% women, mean age: 50.0 ± 10.0 years), all muscle quantity measures were significantly associated with prevalent and incident cardiometabolic risk factors and CVD events. However, only TAM-Fraction remained significantly associated with key outcomes (e.g., adj. OR 0.68 [0.55, 0.84] and HR 0.73 [0.57, 0.92] for incident hypertension and CVD events, respectively) after adjustment for age, sex, body mass index, and waist circumference. Moreover, only higher TAM-Fraction was associated with a lower risk (e.g., adj. OR: 0.56 [0.36–0.89] for incident diabetes versus TAM-Area: adj. OR 1.26 [0.79–2.01] and TAM-Index: 1.09 [0.75–1.58]).

**Conclusion:**

TAM-Fraction on a single CT slice at L3/L4 is a novel body composition marker of cardiometabolic risk in a primary prevention setting that has the potential to improve risk stratification beyond traditional measures of obesity.

## Introduction

Cardiovascular disease (CVD) is the leading cause of death in the USA and worldwide [1]. Physical inactivity is associated with cardiometabolic risk factors and is highlighted as one of the key modifiable risk factors for CVD in the current primary prevention guidelines [2]. Because reliable quantification of daily routine physical activity is challenging, measures of body composition, including obesity and muscle mass, have been proposed as possible objective surrogate markers because they closely associate with the level of physical activity and cardiorespiratory fitness [3, 4].

However, in clinical research, muscle mass has been evaluated predominantly in the context of sarcopenia in patients with chronic diseases, such as chronic lung disease, valvular heart disease, or cancer, demonstrating an association of lower muscle mass with increased morbidity and mortality [5 – 8]. For example, among 2115 obese patients with solid tumors of the respiratory and gastrointestinal tract, low muscle mass on CT was associated with a fourfold increase in mortality [9]. These findings have been explained by muscle wasting due to cancer or chronic disease but are considered a separate matter from cardiovascular effects. In the cardiovascular area, high muscle mass has been predominantly used as a surrogate marker for metabolic fitness, associated with improved prognosis. For instance, in patients with coronary artery disease undergoing percutaneous intervention, those with higher muscle mass showed the lowest risk for mortality and MACE, which is interpreted as a positive effect of higher physical activity for CV outcomes [10].

In contrast to a great body of literature on muscle mass in patients with chronic disease [5 – 9], limited data are available to describe normal distributions of muscle mass in healthy individuals [11]. Low muscle mass on CT in individuals free of chronic disease has been linked to nonalcoholic fatty liver disease [12], and impaired pulmonary function [13]; however, its association with cardiometabolic risk is incompletely understood [11, 14].

Another issue is that no standardized quantitative assessment of TAM exists. The aforementioned include several anatomical locations, including the psoas [5], pectoralis [6], paraspinal [7], and using absolute or relative muscle mass.

To determine whether different measures of abdominal muscle are associated with cardiometabolic risk beyond traditional measures of obesity, we used an established data set of abdominal CT in the community-based Framingham Hearts Study [15, 16] to measure muscle mass as an area on a single CT slice at the level of L3/L4, which represents an easy and straight forward measure. Here, we describe normal distributions of muscle mass in the community and assess the associations of various muscle measures with prevalent and incident cardiometabolic risk factors as well as incident CVD events and propose a standard for muscle mass quantification when assessing its association with CVD risk.

## Materials and methods

### Study cohort

We included participants from the Framingham Heart Study, including the Offspring and 3^rd^-generation cohorts, who underwent routine thoracic and abdominal multidetector computed tomography (MDCT) between 2002 and 2005 [15]. Of the 3,529 participants in the MDCT sub-study, 3,253 were free of artifacts and evaluable for TAM-Area, TAM-Index, and TAM-Fraction. We excluded 237 participants who had prevalent CVD, resulting in a final sample size of 3016 individuals. Each patient gave informed consent and the study protocol was approved by the Institutional Review Board.

### Definition of cardiometabolic risk factors and CVD events

The definition of cardiometabolic risk factors and CVD events in the Framingham Heart Study have been described elsewhere in detail [17]. In short, arterial hypertension was defined as systolic blood pressure ≥ 140 mm Hg or diastolic blood pressure ≥ 90 mm Hg or antihypertensive medication, and diabetes as a fasting plasma glucose level ≥ 126 mg/dL or treatment with either insulin or a hypoglycemic agent. Metabolic syndrome was determined according to the definition of Modified National Cholesterol Education Program Adult Treatment Panel III guidelines [18]. Body mass index (BMI), as a measure of general adiposity, was defined as the weight (kilograms) divided by height squared (meters), and waist circumference (WC), a measure of central adiposity, was measured at the level of the umbilicus.

The physical activity index, a composite score, was calculated by adding the weighted time spent in each activity as described previously [19].

We defined incident CVD as fatal or nonfatal myocardial infarction, fatal or nonfatal stroke, angina, atherothrombotic infarction of the brain, intermittent claudication, heart failure, or CVD death.

### Computed tomography and quantification of muscle mass

**Fig. 1:**
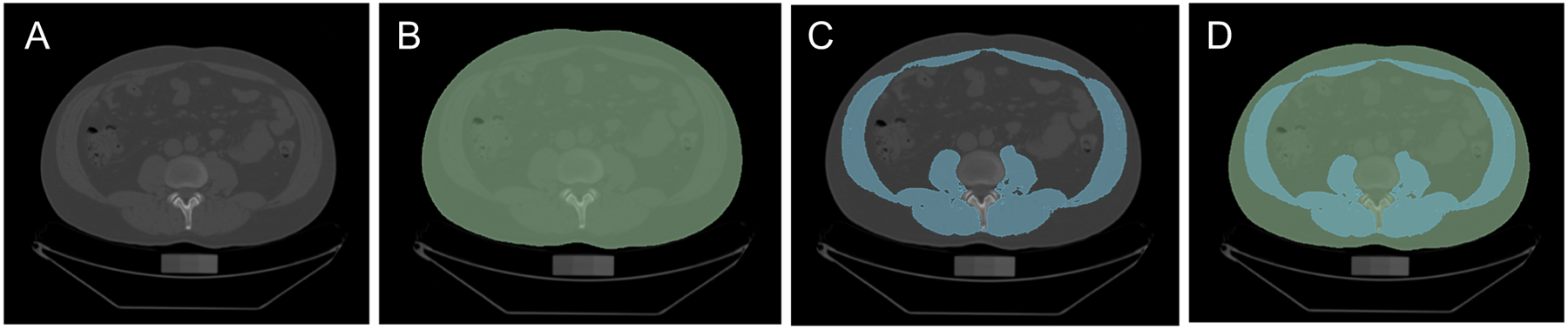
After identification of the slice L3/L4 (panel **A**) and definition of the patient’s cross-sectional area (CSA, green, panel **B**), we used a signal intensity-based threshold approach (−50 to 150 HU) to identify muscle mass (blue; panel **C**). Total abdominal muscle mass fraction (TAM-F) represents the area of muscle mass divided by the CSA (panel **D**)

The technical imaging details have been extensively described elsewhere [15, 20]. In short, participants underwent 8-slice MDCT imaging of the abdomen and 25 continuous 5-mm thick slices were recorded, covering 125 mm above the level of S1 vertebral level. We used the open-access software “3D Slicer,” version 4.8.1 [21], to quantify muscle area on a single slice at the level of L3/L4 based on previous reports [22]. This software allows for easy segmentation processes and offers macro-based solutions for fast imaging post-processing (see Fig. 1).

The abdominal slice at the level L3/L4 was identified manually and each slice was checked for artifacts. Only slices without any artifacts were used for analysis. Muscle was defined based on signal intensity (−50 to 150 HU).

We report three different CT measures of muscle mass, including (A) TAM area (TAM-Area), giving the area of muscle on the single L3/L4 slice in a square centimeter, (B) TAM-Index, indexing TAM-Area to the individual’s height squared, and (C) TAM-Fraction, which is the percentage of TAM in relation to the entire cross-sectional area at the level of L3/L4.

### Statistical analysis

We report mean and standard deviation (SD) and total numbers and percentages for descriptive statistics.

Linear, logistic, and Cox proportional hazards regression were performed to assess the associations of measures of muscle mass (TAM-Area, TAM-Index, and TAM-Fraction) and the following: (A) prevalent cardiometabolic risk factors, including systolic blood pressure (SBP), fasting glucose, cholesterol levels, triglyceride levels, Framingham Risk Score, hypertension, diabetes, and metabolic syndrome; (B) incident cardiometabolic risk factors (hypertension, diabetes, metabolic syndrome), and C) incident CVD. Cross-sectional comparisons were performed at the baseline exam at the time of the CT. For models predicting incident cardiometabolic risk factors, participants who had a prevalent cardiometabolic risk factor of interest at or before baseline were excluded.

To allow for better comparison between different measures of muscle mass, beta coefficients (adj. beta), odds ratios (adj. OR), and adjusted hazard ratios (adj. HR) are reported per a 1-SD increase for each muscle mass measurement.

All regression models were performed (A) age- and sex-adjusted, and (B) age-, sex-, BMI-, and WC-adjusted.

We furthermore report age- and sex-adjusted Pearson correlation coefficients to describe the association between measures of muscle mass with anthropometric measurements, including BMI and WC, and established risk factors, including the Framingham Risk Score.

A two-sided alpha level of 0.05 or less was considered statistically significant. All statistical analyses were performed using SAS v9.4.

## Results

### Study population

A total of 3016 participants (1088 Offspring and 1928 third-generation cohorts) free of CVD were analyzed. Table 1 lists the baseline characteristics of the entire study cohort. The mean age was 50.0 ± 10.0 years and 1472 (48.8%) were women. Prevalence of arterial hypertension, diabetes, and metabolic syndrome was 26.3%, 4.9%, and 24.3%, respectively. The mean BMI was 27.4 kg/m^2^ (SD = 4.8) and a total of 25.4% were labeled as obese based on a BMI of ≥ 30 kg/m^2^.

Mean TAM-Area was 163.5 ± 43.5 cm^2^, ranging from 78.7 to 333.5 cm^2^. When indexed to height in m^2^, TAM-Index was 55.7 ± 11.2 cm^2^/m^2^ (range 29.6–107.8), and TAM-Fraction was 0.24 ± 0.06 (range 0.11–0.51).

### Association of muscle mass with anthropometric measures

**Fig. 2:**
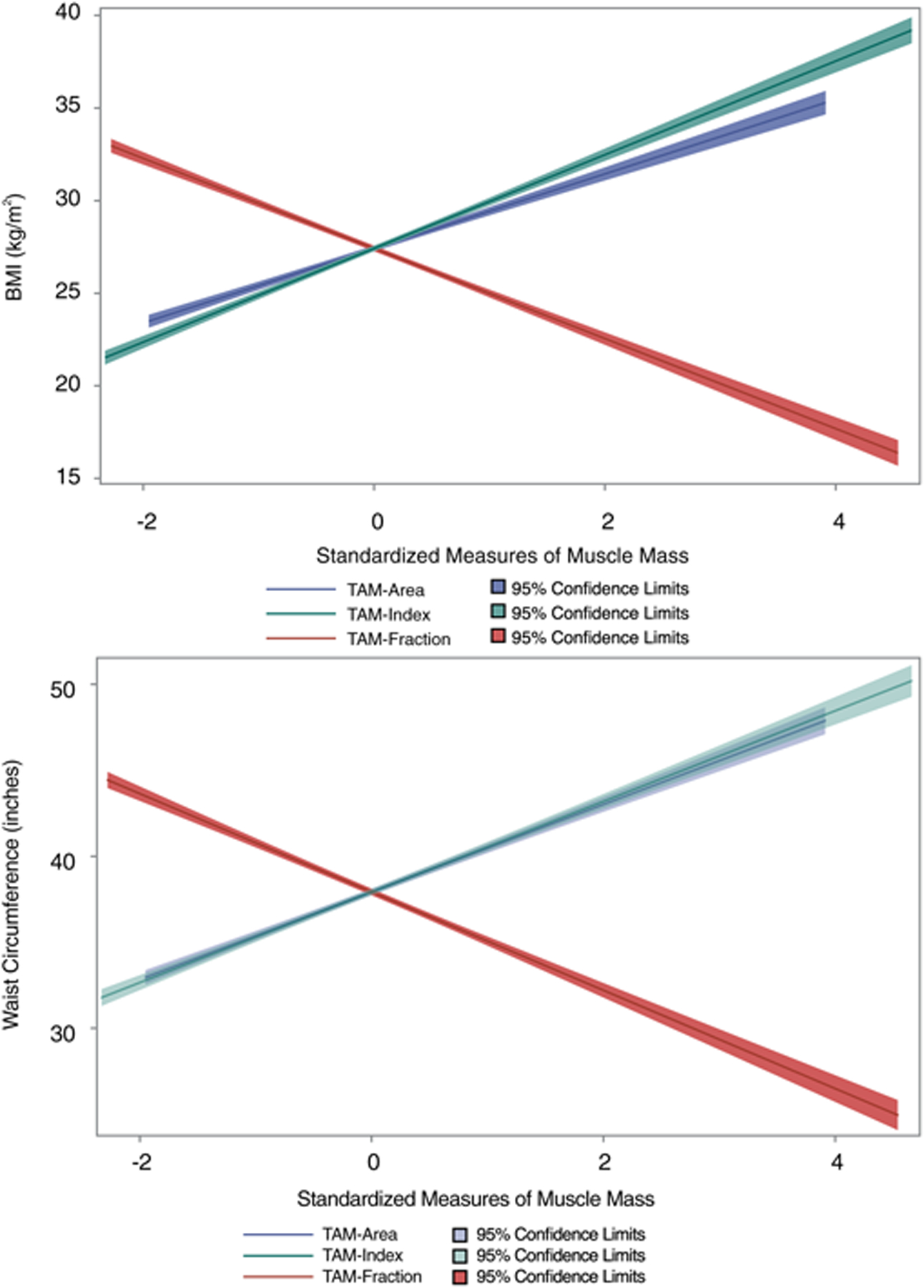
Linear prediction plots (standardized, including 95% confidence interval) demonstrating the opposite direction of association of total abdominal muscle area, index, and fraction (TAM-Area, TAM-Index, TAM-Fraction) with body mass index (BMI, upper panel) and waist circumference (WC, lower panel)

There was a significant correlation between all measures of CT muscle mass and BMI and WC (Table 2, *p* < 0.0001 for all; Fig. 2). After adjustment for age and sex, TAM-Area and TAM-Index were positively correlated with BMI (*r* = 0.578 and 0.621, *p* < .0001 each) and WC (*r* = 0.470 and 0.420, *p* < .0001 each). In contrast, TAM-Fraction was negatively correlated with both anthropometric measures of adiposity (*r* = −0.655 and −0.662, for BMI and WC respectively; *p* < .0001 for both).

Table 3 depicts differences in TAM-Area, TAM-Index, and TAM-Fraction between individuals without and with BMI indicative of obesity. Individuals with obesity had significantly higher TAM-Area (182.9 ± 45.3 versus 156.9 ± 40.8 kg/m^2^, *p* < .0001) and TAM-Index (62.5 ± 11.4 versus 53.4 ± 10.2 cm^2^/m^2^, *p* < .0001) but lower TAM-Fraction (0.20 ± 0.04 versus 0.26 ± 0.06, *p* < .0001) as compared to individuals without obesity. We observed the same pattern when comparing individuals with and without hypertension and diabetes. Patients with hypertension and diabetes had higher TAM-Area and TAM-Index but lower TAM-Fraction when compared to their disease-free counterparts (details, see Table 3).

### Association of muscle mass with prevalent cardiometabolic risk factors

TAM-Area, TAM-Index, and TAM-Fraction were significantly associated with prevalent cardiometabolic risk (Tables 4 and 5). However, the directionality of the association was substantially different between the measures. In general, higher TAM-Area and TAM-Index were associated with an altered risk profile (e.g., for TAM-Area: adj. Beta 3.81 [95%CI: 2.67, 4.96], *p* < .0001 for systolic blood pressure; 5.09 [95%CI: 3.86, 6.31], *p* < .0001 for fasting glucose, per 1-SD increase after adjustment for age and sex respectively) and higher prevalence of cardiometabolic risk factors (e.g., for TAM-Index: adj. OR 1.65 [95%CI: 1.45,1.87] for hypertension, and 1.72 [95%CI: 1.37, 2.15] for diabetes, *p* < .0001 each, per 1-SD increase, age- and sex-adjusted).

In contrast, higher TAM-Fraction was associated with significantly more favorable risk profile (e.g., adj. Beta −4.16 [95% CI: −4.86, −3.46], *p* < .0001 for systolic blood pressure, per 1-SD increase, adjusted for age and sex) and lower prevalence of hypertension, diabetes, and metabolic syndrome (adj. OR 0.52 [95%CI: 0.46, 0.59], 0.45 [95%CI: 0.35, 0.59], and 0.36 [95%CI: 0.32, 0.41], *p* < .0001 for all, adjusted for age and sex).

After further adjustment for anthropometric measures of overall and central adiposity (BMI and WC), TAM-Area and TAM-Index were not significantly associated with key risk factors (see Table 2). However, higher TAM-Fraction remained significantly associated with a more favorable risk profile (e.g. adj. Beta −1.54 [−2.50, −0.58], *p* = 0.0017 for systolic blood pressure, per 1-SD increase) and a lower prevalence of hypertension and metabolic syndrome (adj. OR 0.74 [95%CI: 0.63, 0.87], *p* = 0.0002 and 0.76 [95%CI: 0.63, 0.90], *p* = 0.0015).

### Association with incident cardiometabolic risk factors

During 6.4 years of follow-up, a total of 192 (7.0%) incident cases of diabetes were observed, as were 1,005 (36.4%) of hypertension, and 825 (30.5%) of metabolic syndrome.

The association between measures of muscle mass with incident cardiometabolic events is displayed in Table 6. Similar to the association with prevalent cardiometabolic risk, in age-and sex-adjusted analyses, higher TAM-Area and TAM-Index were associated with a higher risk for developing hypertension, diabetes, and CVD. In contrast, higher TAM-Fraction was protective against all these incident findings. After further adjustment for BMI and WC, only TAM-Fraction was significantly associated with lower risk for incident outcomes (adj. OR 0.68 [95%CI: 0.55, 0.84] for hypertension, 0.57 [95%CI: 0.36, 0.89] for diabetes, *p* < 0.05 each; trend for metabolic syndrome: 0.80 [95%CI: 0.64, 1.00], *p* = 0.053). Of note, most associations were not significant for TAM-Area and TAM-Index except for the higher risk of incident metabolic syndrome for TAM-Index (adj. OR 1.28 [95%CI: 1.00, 1.63], *p* = 0.049).

### Association with CVD events

During 11 years of follow-up, a total of 244 CVD events were recorded. In age- and sex-adjusted analysis, a similar trend as with incident cardiometabolic risk factors was observed, with a higher risk for events with TAM-Area (adj. HR 1.35 [95%CI: 1.06, 1.72], *p* = 0.015) and TAM-Index (adj. HR 1.25 [95%CI: 1.05, 1.50], *p* = 0.014) but a lower risk for TAM-Fraction (adj. HR 0.64 [0.53, 0.76], *p* < .0001). When additionally adjusted for BMI and WC, only higher TAM-Fraction remained significantly associated with a lower risk for CVD events (adj. HR 0.73 [95%CI: 0.57, 0.92], *p* = 0.009).

**Fig. 3:**
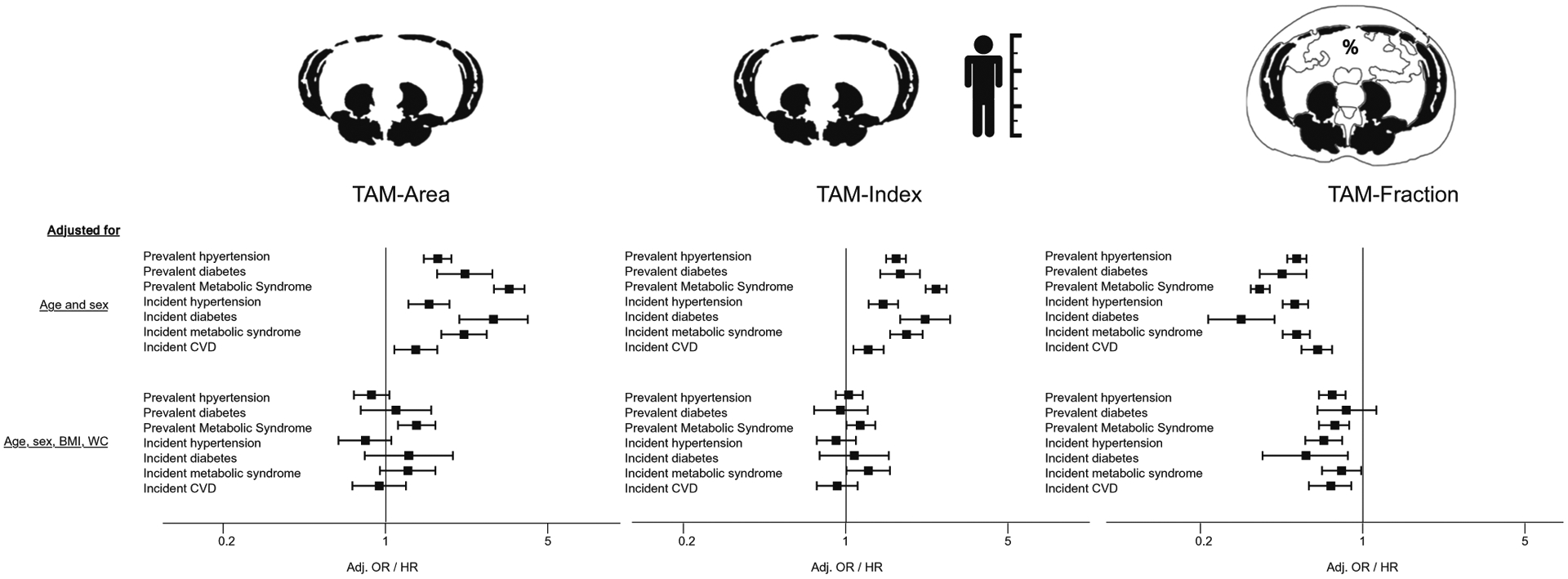
Association of total abdominal muscle area, index, and fraction (TAM-Area, TAM-Index, and TAM-Fraction) with prevalent and incident cardiometabolic risk factors and cardiovascular disease (CVD) events. Odds and hazard ratios are given per 1-SD increase of TAM-Area, TAM-Index, and TAM-Fraction respectively

Figure 2 displays the opposite direction of association between TAM-Area and TAM-Index, compared with TAM-Fraction, with BMI and WC. Figure 3 displays the different directionality between TAM-Area, TAM-Index, and TAM-Fraction with prevalent and incident outcomes.

## Discussion

In a community-based cohort of participants free of CVD, muscle mass assessed on a single-slice abdominal CT scan associates with cardiometabolic and CVD risk. However, among different CT measures of muscle mass, only for TAM-Fraction (less muscle) was associated with higher cardiometabolic risk, suggesting that further investigations defining a new obesity phenotype to improve risk stratification may primarily focus on TAM-Fraction among the various ways to measure muscle.

Physical activity and avoidance of excessive adipose tissue play an important part in cardiometabolic disease prevention. This is highlighted in the 2019 ACC/AHA Guideline on the Primary Prevention of Cardiovascular Disease, which promotes at least 150 min per week of accumulated moderate-intensity physical activity or 75 min per week of vigorous-intensity physical activity [2]. This recommendation targets primarily the avoidance of obesity primarily as a key risk factor for cardiometabolic risk, similar to the role of coronary artery calcification as a risk factor for CVD [2]. However, standardized quantification of physical activity is challenging as self-reported data lack accuracy [23, 24] and functional assessment of cardiorespiratory fitness is not feasible in a population-based setting [25].

Assessment of BMI as a marker of general adiposity is easy and recommended annually in all adults and WC as an indicator for central obesity in individuals at higher cardiometabolic risk [2]. In contrast, no standardized measure is available for muscle mass, another key body composition component besides obesity. Muscle mass has been shown to correlate with the level of physical activity [3]. It can easily be assessed and quantified on computed tomography (CT) and magnetic resonance imaging (MRI), commonly used imaging techniques for various indications. In chronic disease, muscle mass on CT as a surrogate for physical fitness is well established. Low muscle mass—interpreted as a disease-associated loss of function—is associated with worse outcomes. This has been shown for patients with lung disease [26], valvular heart disease [5], coronary artery disease [10], and especially cancer [27].

In contrast to patients with chronic disease, only a few studies report on muscle mass in population-based settings [12, 13] and its association with CV risk is unknown.

Several factors may contribute to the fact that muscle mass is not clinically used as a body composition component for cardiometabolic risk assessment, including a lack of a standardized approach to assess and report muscle mass. Different anatomic landmarks have been proposed, including the psoas muscle [5], the pectoralis muscle [6], paraspinal muscles [7], or skeletal muscle mass at the thigh level [8]. This substantially hampers direct comparison between studies. In our study, we used a standardized approach to quantify muscle mass at the level of L3/L4 on a single abdominal CT slice. This has been shown to correlate almost perfectly with volumetric data of adipose tissue in the Framingham Heart Study [22].

Furthermore, some authors report total muscle area (TAM-Area) on a single or multiple tomographic slices [28, 29], whereas others index muscle mass to body height or body surface area (TAM-Index) [5, 12]. We chose to measure both not-indexed TAM-Area as well as indexed to height because these were reported in previous studies [5, 12, 28, 29]. In our cohort, we provide for the first time descriptive data for TAM-Area and TAM-Index and, in addition, introduce the concept of TAM-Fraction as a novel body composition tool, representing the fraction of muscle mass of the entire tomographic L3/L4 slice. Comparing these three measures of muscle mass, we observed a different directionality in the associations of these measures with the risk of outcome events. Higher TAM-Area and TAM-Index were associated with a worse cardiometabolic risk profile in age and sex-adjusted models. Similar findings are reported by an analysis of the Multi-Ethnic Study of Atherosclerosis (MESA), where higher TAM-Area, averaged from several single slices between L2 and L5, was associated with more CAC burden. This was in contrast to the authors’ hypothesis of a protective effect of higher muscle mass [29] but is similar to our results for higher TAM-Area and TAM-Index indicating higher cardiometabolic risk. This might, in part, be explained by a close positive correlation between TAM-Area and TAM-Index and both BMI and WC.

In contrast, TAM-Fraction is negatively correlated with BMI and WC. Indeed, higher TAM-Fraction indicated a beneficial cardiometabolic risk profile across a wide spectrum of prevalent and incident risk factors. Higher TAM-Fraction, but not TAM-Area or TAM-Index, remained significantly associated with lower prevalence of several cardiometabolic risk factors, including hypertension and metabolic syndrome, and lower risk for new-onset hypertension and diabetes and CVD events even when adjusted for BMI and WC.

While the purpose of this paper was to introduce the concept of TAM-Fraction as a novel body composition tool, representing the fraction of muscle mass of the entire tomographic L3/L4 slice, future studies are warranted to investigate the interplay between TAM-Fraction and more advanced measures of adiposity, including visceral and subcutaneous adipose tissue, with the potential to define a more precise individual obesity phenotype for improved CV risk prediction.

## Limitations

Several limitations merit comment. As with any analyses from the Framingham Heart Study, we were not able to assess ethnic/race-related differences due to our predominant white study sample. In addition, CT scans were performed between 2002 and 2005, when demographic characteristics, such as obesity rates, were different compared to the present [30]. This analysis also lacks information on fat infiltration within the skeletal muscle, which may be an important aspect.

## Figures and Tables

**Table 1:** Baseline characteristics participants free of prevalent cardiovascular disease

Variable	All participants* (*N* = 3016)
Demographics and anthropometrics	
Age - years	50.0 (10.0)
Female sex	1472 (48.8)
Body weight - kg	78.7 (16.1)
Body height - m	1.7 (0.1)
BMI - kg/m^2^	27.4 (4.8)
Waist circumference - inches	37.9 (6.0)
Risk factors	
Hypertension	793 (26.3)
Use of antihypertensive drugs	483 (16.0)
Systolic blood pressure – mm Hg	121 (18)
Diastolic blood pressure – mm Hg	77 (21)
Diabetes	148 (4.9)
Fasting glucose - mmol/L	98 (18)
Total cholesterol - mmol/L	197 (35)
HDL cholesterol - mmol/L	54 (17)
LDL cholesterol - mmol/L	118 (31)
Triglycerides, mmol/L	124 (87)
Use of lipid-lowering drugs	345 (11.4)
Current smoking	382 (12.7)
Physical activity index	37.57 (7.32)
Framingham risk score	0.09 (0.09)
ASCVD risk score	0.06 (0.09)
CT Muscle mass parameters	
TAM-Area – cm^2^	163.5 (43.5)
TAM-Index – cm^2^/m^2^	55.7 (11.2)
TAM-Fraction - (%)	0.24 (0.06)

* Values are mean (SD) for continuous variables and % for categorical ones

*BMI* indicates body mass index; *HDL*, high-density lipoprotein; *LDL*, low-density lipoprotein; *ASCVD*, Atherosclerotic Cardiovascular Disease; *TAM*, total abdominal muscle

**Table 2:** Correlation analyses between total abdominal muscle area, index, and fraction (TAM-Area, TAM-Index, TAM-Fraction) with conventional measures of adiposity and cardiovascular risk scores

Variable	Age and sex adjusted					
	TAM-Area		TAM-Index		TAM-Fraction	
	Rho	*p*	Rho	*p*	Rho	*p*
BMI	0.5780	< .0001	0.6212	< .0001	−0.6552	< .0001
Waist circumference	0.4695	< .0001	0.4197	< .0001	−0.6615	< .0001
Framingham risk Score	0.0910	< .0001	0.1274	< .0001	−0.1614	< .0001
ASCVD risk score	0.0737	< .0001	0.1106	< .0001	−0.0659	0.0003

*BMI* indicates body mass index; *ASCVD*, atherosclerotic cardiovascular disease; *TAM*, total abdominal muscle

**Table 3:** Mean values of total abdominal muscle area, index, and fraction (TAM-Area, TAM-Index, TAM-Fraction) when stratified by the presence of obesity, hypertension, and diabetes

Variable	Statistics	Obesity		*p*	No (73.7%)	Hypertension	*p*	Diabetes		*p*
		No (74.6%)	Yes (25.4%)			Yes (26.3%)		No (95.1%)	Yes (4.9%)	
TAM-Area	Mean (S.D.)	156.87 (40.79)	182.91 (45.26)	<.0001	162.24 (43.04)	166.93 (44.45)	0.0091	162.99 (43.34)	172.89 (44.78)	0.0068
TAM-Index	Mean (S.D.)	53.40 (10.15)	62.46 (11.36)	<.0001	55.08 (11.00)	57.43 (11.50)	<.0001	55.54 (11.13)	58.69 (11.73)	0.0009
TAM-Fraction	Mean (S.D.)	0.26 (0.06)	0.20 (0.04)	<.0001	0.25 (0.06)	0.22 (0.05)	<.0001	0.24 (0.06)	0.21 (0.05)	< .0001

*TAM* indicates total abdominal muscle

**Table 4:** Association total abdominal muscle area, index, and fraction (TAM-Area, TAM-Index, TAM-Fraction) with prevalent cardiometabolic risk factors

Outcome	Predictor	Model A*		Model B**	
		Adjusted Beta per 1SD Increase	*p*	Adjusted Beta per 1SD Increase	*p*
SBP	TAM-Area	3.81 (2.67, 4.96)	< .0001	−1.05 (−2.42, 0.32)	0.1328
	TAM-Index	3.46 (2.60, 4.32)	< .0001	−0.11 (−1.21, 1.00)	0.8496
	TAM-Fraction	−4.16 (−4.86, −3.46)	< .0001	−1.54 (−2.50, −0.58)	0.0017
DBP	TAM-Area	2.00 (0.56, 3.43)	0.0063	−0.41 (−2.17, 1.34)	0.6466
	TAM-Index	1.67 (0.59, 2.75)	0.0025	−0.31 (−1.72, 1.11)	0.6718
	TAM-Fraction	−2.08 (−2.97, −1.19)	< .0001	−0.91 (−2.14, 0.32)	0.1471
Fasting glucose	TAM-Area	5.09 (3.86, 6.31)	< .0001	0.39 (−1.07, 1.85)	0.6007
	TAM-Index	3.95 (3.03, 4.87)	< .0001	0.30 (−0.88, 1.48)	0.6160
	TAM-Fraction	−3.85 (−4.60, −3.10)	< .0001	−0.43 (−1.45, 0.60)	0.4123
Total cholesterol	TAM-Area	2.57 (0.16,4.97)	0.0362	−0.53 (−3.46, 2.41)	0.7260
	TAM-Index	4.57 (2.77, 6.37)	< .0001	4.02 (1.66, 6.38)	0.0008
	TAM-Fraction	−4.34 (−5.83, −2.86)	< .0001	−4.06 (−6.12, −2.01)	0.0001
HDL cholesterol	TAM-Area	−5.79 (−6.79, −4.79)	< .0001	−1.22 (−2.41, −0.03)	0.0448
	TAM-Index	−4.03 (−4.79, −3.27)	< .0001	−0.21 (−1.18,0.75)	0.6656
	TAM-Fraction	3.90 (3.28, 4.52)	< .0001	0.42 (−0.42, 1.26)	0.3262
LDL cholesterol	TAM-Area	3.72 (1.56, 5.88)	0.0008	0.18 (−2.47, 2.83)	0.8937
	TAM-Index	4.77 (3.14,6.39)	< .0001	3.40 (1.28, 5.52)	0.0017
	TAM-Fraction	−4.03 (−5.35, −2.70)	< .0001	−2.67 (−4.51, −0.84)	0.0043
Log triglycerides	TAM-Area	25.31 (19.49,31.12)	< .0001	3.84 (−3.17, 10.84)	0.2828
	TAM-Index	20.67 (16.30,25.03)	< .0001	5.31 (−0.32, 10.93)	0.0646
	TAM-Fraction	−22.50 (−26.07, −18.94)	< .0001	−10.30 (−15.20, −5.39)	< .0001
Physical activity index	TAM-Area	0.56 (0.05, 1.06)	0.0319	1.37 (0.75, 1.99)	< .0001
	TAM-Index	0.72 (0.33, 1.10)	0.0003	1.53 (1.03, 2.03)	< .0001
	TAM-Fraction	0.82 (0.50, 1.13)	< .0001	0.85 (0.41, 1.29)	0.0002
Framingham risk score	TAM-Area	0.01 (0.01, 0.02)	< .0001	−0.00 (−0.01, 0.00)	0.1246
	TAM-Index	0.01 (0.01, 0.01)	< .0001	0.00 (−0.00, 0.01)	0.7097
	TAM-Fraction	−0.01 (−0.02, −0.01)	<.0001	−0.00 (−0.01, −0.00)	0.0332

*SBP* indicates systolic blood pressure; *DBP*, diastolic blood pressure; *HDL*, high-density lipoprotein; *LDL*, low-density lipoprotein.

* Model A adjusts for age and sex

** Model B adjusts for age, sex, BMI, and WC

**Table 5:** Association total abdominal muscle area, index, and fraction (TAM-Area, TAM-Index, TAM-Fraction) with prevalent hypertension, diabetes, and metabolic syndrome

Outcome	Predictor	Model A*		Model B**	
		Adjusted OR per 1 SD Increase	*p*	Adjusted OR per 1 SD Increase	*p*
Prevalent hypertension	TAM-Area	1.68 (1.42, 1.98)	< .0001	0.87 (0.71, 1.07)	0.1988
	TAM-Index	1.65 (1.45, 1.87)	< .0001	1.03 (0.88, 1.22)	0.7042
	TAM-Fraction	0.52 (0.46, 0.59)	< .0001	0.74 (0.63, 0.87)	0.0002
Prevalent diabetes	TAM-Area	2.20 (1.62, 2.97)	< .0001	1.11 (0.76, 1.62)	0.5818
	TAM-Index	1.72 (1.37, 2.15)	< .0001	0.95 (0.71, 1.28)	0.7464
	TAM-Fraction	0.45 (0.35, 0.59)	< .0001	0.85 (0.62, 1.18)	0.3398
Prevalent metabolic syndrome	TAM-Area	3.41 (2.85, 4.09)	< .0001	1.36 (1.10, 1.69)	0.0051
	TAM-Index	2.45 (2.15, 2.80)	< .0001	1.18 (1.00, 1.41)	0.0543
	TAM-Fraction	0.36 (0.32, 0.41)	< .0001	0.76 (0.63, 0.90)	0.0015

* Model A adjusts for age and sex

** Model B adjusts for age, sex, BMI, and WC

**Table 6:** Logistic regression model demonstrating the association between total abdominal muscle area, index, and fraction (TAM-Area, TAM-Index, TAM-Fraction) and incident cardiometabolic risk factors as well as cardiovascular disease (CVD) events

Outcome		Model A*	Model B**		
	Predictor	Adjusted OR or HR per 1SD increase	*p*	Adjusted OR or HR per 1SD increase	*p*
Incident hypertension	TAM-Area	1.54 (1.22, 1.94)	0.0003	0.82 (0.61, 1.09)	0.1739
	TAM-Index	1.45 (1.22, 1.73)	< .0001	0.91 (0.73, 1.14)	0.4244
	TAM-Fraction	0.51 (0.44, 0.60)	< .0001	0.68 (0.55, 0.84)	0.0004
Incident diabetes	TAM-Area	2.92 (2.02, 4.22)	< .0001	1.26 (0.79, 2.01)	0.3251
	TAM-Index	2.20 (1.67, 2.90)	< .0001	1.09 (0.75, 1.58)	0.6447
	TAM-Fraction	0.30 (0.21, 0.43)	< .0001	0.57 (0.36, 0.89)	0.0139
Incident metabolic syndrome	TAM-Area	2.18 (1.69, 2.81)	< .0001	1.25 (0.92, 1.69)	0.1556
	TAM-Index	1.83 (1.51,2.21)	< .0001	1.28 (1.00, 1.63)	0.0486
	TAM-Fraction	0.52 (0.44, 0.61)	< .0001	0.80 (0.64, 1.00)	0.0533
Incident CVD	TAM-Area	1.35 (1.06, 1.72)	0.0153	0.94 (0.70, 1.26)	0.6825
	TAM-Index	1.25 (1.05, 1.50)	0.0142	0.92 (0.73, 1.16)	0.4615
	TAM-Fraction	0.64 (0.53, 0.76)	< .0001	0.73 (0.57, 0.92)	0.0094

*CVD* indicates cardiovascular disease

* Model A adjusts for age and sex

** Model B adjusts for age, sex, BMI, and WC

## Key Points

- In this analysis of the Framingham Heart Study (n = 3016), TAM-F on a single slice CT was more closely associated with prevalent and incident cardiometabolic risk factors as compared to TAM alone or TAM indexed to body surface area.
- TAM-F on a single abdominal CT slice at the level of L3/L4 could serve as a standard measure of muscle mass and improve risk prediction

## Abbreviations

BMI: Body mass index
CSA: Cross-sectional area
CVD: Cardiovascular disease
TAM: Total abdominal muscle mass
TAM-A: Total abdominal muscle area
TAM-F: Total abdominal muscle fraction
TAM-I: Total abdominal muscle index
WC: Waist circumference
